# Design, Synthesis, In Vitro, and In Silico Studies of New *N*^5^-Substituted-pyrazolo[3,4-*d*]pyrimidinone Derivatives as Anticancer CDK2 Inhibitors

**DOI:** 10.3390/ph16111593

**Published:** 2023-11-11

**Authors:** Waheed A. Zaki, Selwan M. El-Sayed, Mohamed Alswah, Ahmed El-Morsy, Ashraf H. Bayoumi, Abrahman S. Mayhoub, Walaa H. Moustafa, Aeshah A. Awaji, Eun Joo Roh, Ahmed H.E. Hassan, Kazem Mahmoud

**Affiliations:** 1 Department of Pharmaceutical Organic Chemistry, Faculty of Pharmacy, Al-Azhar University, Cairo 11884, Egypt; 2 Department of Medicinal Chemistry, Faculty of Pharmacy, Mansoura University, Mansoura 35516, Egypt; 3 Pharmaceutical Chemistry Department, College of Pharmacy, The Islamic University, Najaf 54001, Iraq; 4 Nanoscience Program, University of Science and Technology, Zewail City of Science and Technology, October Gardens, 6th of October, Giza 12578, Egypt; 5 Microbiology and Immunology Department, Faculty of Pharmacy, Helwan University, Cairo 19448, Egypt; 6 Department of Biology, Faculty of Science, University College of Taymaa, University of Tabuk, Tabuk 71491, Saudi Arabia; 7 Chemical and Biological Integrative Research Center, Korea Institute of Science and Technology (KIST), Seoul 02792, Republic of Korea; 8 Division of Bio-Medical Science & Technology, University of Science and Technology, Daejeon 34113, Republic of Korea; 9 Department of Pharmaceutical Chemistry, Faculty of Pharmacy, Egyptian Russian University, Badr City 11829, Egypt

**Keywords:** anticancer agents, liver cancer, colorectal cancer, CDK2, pyrazolo[3,4-*d*]pyrimidine analogs

## Abstract

CDK2 is a key player in cell cycle processes. It has a crucial role in the progression of various cancers. Hepatocellular carcinoma (HCC) and colorectal cancer (CRC) are two common cancers that affect humans worldwide. The available therapeutic options suffer from many drawbacks including high toxicity and decreased specificity. Therefore, there is a need for more effective and safer therapeutic agents. A series of new pyrazolo[3,4-*d*]pyrimidine analogs was designed, synthesized, and evaluated as anticancer agents against the CRC and HCC cells, HCT116, and HepG2, respectively. Pyrazolo[3,4-*d*]pyrimidinone derivatives bearing *N*^5^-2-(4-halophenyl) acetamide substituents were identified as the most potent amongst evaluated compounds. Further evaluation of CDK2 kinase inhibition of two potential cytotoxic compounds **4a** and **4b** confirmed their CDK2 inhibitory activity. Compound **4a** was more potent than the reference roscovitine regarding the CDK2 inhibitory activity (IC_50_ values: 0.21 and 0.25 µM, respectively). In silico molecular docking provided insights into the molecular interactions of compounds **4a** and **4b** with important amino acids within the ATP-binding site of CDK2 (Ile10, Leu83, and Leu134). Overall, compounds **4a** and **4b** were identified as interesting CDK2 inhibitors eliciting antiproliferative activity against the CRC and HCC cells, HCT116 and HepG2, respectively, for future further investigations and development.

## 1. Introduction

Cancer is a leading cause of morbidity and mortality in addition to being a significant barrier to increasing life expectancy worldwide. According to the cancer statistics update (GLOBOCAN 2020) reported by the International Agency for Research on Cancer (IARC), it is estimated that there were 19.3 million new cases of cancer and about 10 million deaths from cancer in 2020 globally [1]. In fact, cancer comprises diverse and heterogenous diseases with different incidence and morbidity rates. While CRC ranks third and liver cancer ranks sixth in regard to global incidence rates, colorectal cancer has the second highest morbidity, while liver cancer has the third highest [2,3,4,5]. In particular, liver cancer is one of the most commonly diagnosed cancers amongst the Egyptian population. Meanwhile, CRC shows an increasing incidence rate amongst the Arab world population [6].

HCC is the predominant form of liver cancer accounting for about 80–85% of liver cancer cases [2]. High recurrence rates and metastases are major hurdles that limit the successfulness of current treatment protocols [7]. Despite the advent of multi-target kinase inhibitors such as sorafenib, lenvatinib, regorafenib, and cabozantinib which increased survival rates of HCC patients, evolvement of resistance and development of metastases are often encountered issues [7,8]. Likewise, current CRC treatments are hampered by the development of metastatic CRC resulting in high mortality rates [9,10]. Moreover, all current chemotherapeutic medications for HCC and CRC suffer from various systemic toxicities, lower rates of successful treatment, and variable innate and acquired resistances in addition to unacceptable decreased tumor specificity. Consequently, HCC and CRC are major challenging health problems that raise urgent needs for the development of new effective treatments. The development of effective and affordable medicines is an important consideration. An effective but prohibitively expensive medicine would be inaccessible to vulnerable patients in poor populations. According to the World Bank census data of 2021, there are 3.4 billion and 700 million people in lower-middle-income and low-income countries, respectively. The cost of active pharmaceutical ingredients is amongst the major determinants of medicine prices and, consequently, affordability. Therefore, development of an economical synthesis method, including step economy and employing inexpensive substances, is important [11,12].

Cyclin dependent kinases (CDKs) belong to a family of serine/threonine kinases that requires a cyclin protein for their action. They have axial regulatory roles in cell progression, differentiation, and apoptosis [13,14,15]. CDKs were found overexpressed in several cancers including HCC and CRC [16,17,18]. Among the identified twenty-one CDKs, CDK2 is a key player in cell cycle progress [19,20]. Cell cycle deregulation associated with CDK2 overexpression is a hallmark of the development of cancerous cells [21]. Accordingly, developing CDK2 inhibitors might be a promising and attractive approach to achieve new anticancer therapeutic agents [22]. Various CDK2 inhibitors are reported and some are in clinical trials as potential anticancer agents such as flavopiridol, dinaciclib, roscovitine, and milciclib [23].

**Figure 1:**
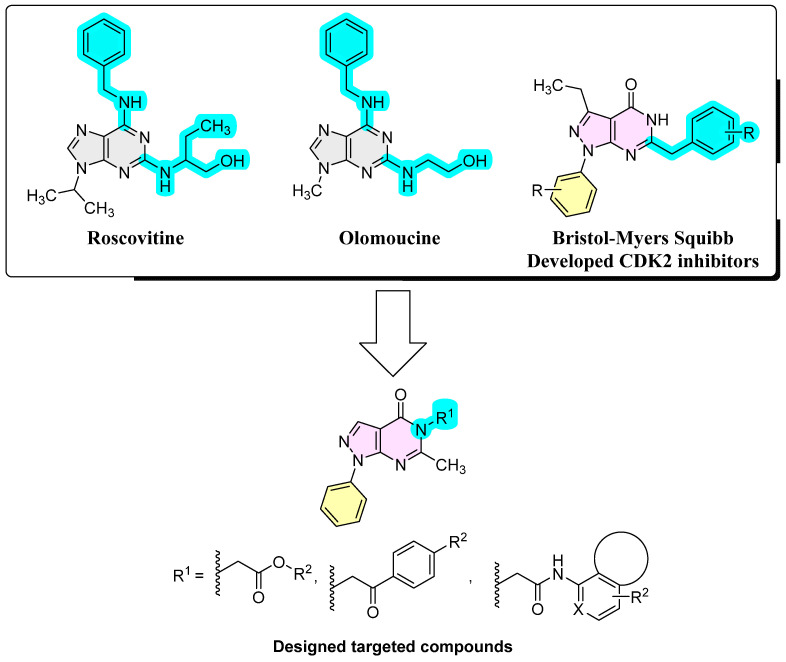
Literature known CDK2 inhibitors and the new designed target compounds.

Cyclin-dependent kinase-2 (CDK2) requires, in addition to cyclin, the adenosine triphosphate (ATP) molecule to function. Knowing that ATP incorporates adenine which is a purine base, it might not be surprising that purine and purine isosteres serve as scaffolds to develop CDK2 inhibitors. Thus, roscovitine and olomoucine (Figure 1) were developed as purine based CDK2 inhibitors [24]. While roscovitine and olomoucine are 4,6-disubstituted purine derivatives, Bristol–Myers Squibb has developed 6-substituted-pyrazolo[3,4-*d*]pyrimidinones with a purine isostere scaffold, as CDK2 inhibitors [25]. Exploration of positional isomers is one of the most well-known drug design strategies and a powerful lead generation method that can yield new chemical entities of promising activity [26,27,28]. While 4,6-disubstituted purine derivatives and 6-substituted-pyrazolo[3,4-*d*]pyrimidinones as CDK2 inhibitors are reported, *N*^5^-substituted-pyrazolo[3,4-*d*]pyrimidinones, to the best of our knowledge, have not been explored as potential CDK2 inhibitors [29,30,31]. Considering these data and motivated by the positional isomerism strategy in generating new promising bioactive compounds, new derivatives of *N*^5^-substituted-pyrazolo[3,4-*d*]pyrimidinones were designed as antiproliferative CDK2 inhibitors against HCC and CRC. As shown in Figure 1, the compounds designed maintained the *N*^1^-aryl substituent existing in Bristol–Myers Squibb-developed compounds but introduced diverse *N*^5^-substituents at the pyrazolo[3,4-*d*]pyrimidinone scaffold while substituents at the 6-position were trimmed, leaving only the small methyl fragment at the 6-position. In the move towards a more efficient exploration of the chemical space around *N*^5^-substituents, diverse substituents were considered involving variable alkyl, acetyl esters, phenacyl or acetamide moieties with diverse substituents and/or stereoelectronic features. Herein, we report our interesting results of the designed compounds.

## 2. Results and Discussion

### 2.1. Chemistry

**Scheme 1:**
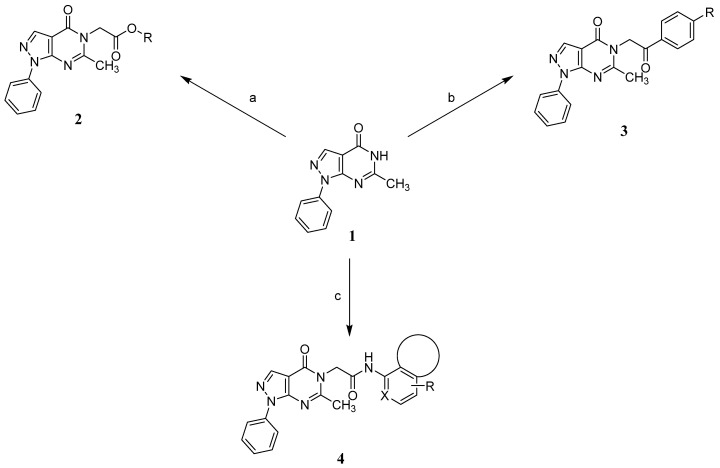
Synthetic routes to access the desired compounds: (a) appropriate 2-chloroacetate ester derivative, K_2_CO_3_, DMF, 100 °C, 5–24 h, 75% yield; (b) appropriate 2-bromoacertophenone derivative, K_2_CO_3_, DMF, 100 °C, 5–24 h, 62–65% yield; and (c) appropriate 2-chloroacetamide derivative, K_2_CO_3_, DMF, 100 °C, 5–24 h, 60–70% yield.

A concise synthesis of the targeted compounds was achieved in one single step starting from the reported 1,5-dihydro-4*H*-pyrazolo[3,4-*d*]pyrimidin-4-one derivative **1** [32]. As shown in Scheme 1, *N*^5^-alkylation using the appropriate halo derivative in the presence of a potassium carbonate/DMF system directly afforded the corresponding targeted compounds. Thus, heating a mixture of compound **1** with the appropriate 2-chloroacetate ester derivative in the presence of a potassium carbonate/DMF system afforded the desired derivative **2** at a 75% yield. Similarly, heating a mixture of compound **1** with the appropriate 2-bromoacertophenone derivative in the presence of a potassium carbonate/DMF system afforded the desired derivative **3** at 62–65% yields. Likewise, heating a mixture of compound **1** with the appropriate 2-chloroacetamide derivative in the presence of a potassium carbonate/DMF system afforded the desired derivative **4** at 60–70% yields.

### 2.2. In Vitro Antiproliferative Activity

Towards the evaluation of the activity of synthesized compounds against CRC and HCC, HCT116 cells (a CRC cell line) and HepG2 cells (an HCC cell line) were used, adopting the colorimetric MTT assay protocol, which is well-known in the literature [33,34]. To assess the results, the activity of the evaluated compounds was compared to the reference CDK2 inhibitor, roscovitine [35]. In addition, the most active compounds in this assay were further tested against the normal human fibroblast cell line (WI-38) in order to assess their cytotoxic effect on normal cells. The assay outcome is summarized in Table 1.

The reference standard roscovitine inhibited the growth of the employed CRC and HCC cells showing moderate IC_50_ values of 12.24 and 13.82 µM against HCT116 and HepG2 cells, respectively. Amongst the evaluated compounds, two *N*^5^-2-acetamide derivatives, **4a** and **4b**, showed interestingly superior activity to the reference roscovitine standard against both the HCT116 and HepG2 cells. Both compounds **4a** and **4b** were more active than roscovitine against HepG2 cells and HCT116 cells. Compound **4a** was almost twenty-four- and eleven-fold more potent than roscovitine against HepG2 and HCT116, respectively (IC_50_ of 0.58 and 1.09 µM for compound **4a**). Meanwhile, compound **4b** was almost 5.3- and 3.6-fold more potent than roscovitine against HepG2 and HCT116, respectively (IC_50_ of 2.57 and 3.38 µM for compound **4b**). Despite the *N*^5^-phenacyl derivative **3b** showing moderate activity against HCT116 similar to roscovitine (IC_50_ of 10.75 µM versus 12.24 µM, respectively), it was similar to compounds **4a** and **4b** in maintaining the same activity trend of higher activity against HepG2 cells, showing nearly seven-fold higher potency relative to roscovitine. Interestingly, the *N*^5^-phenacyl derivative **3a** and *N*^5^-2-acetamide derivative **4e** maintained a similar activity trend where they were more active against HepG2 relative to HCT116. Thus, compounds **4e** and **3a** showed intermediate activities against HepG2 cells with determined IC_50_ values of 16.08 and 26.25 µM, respectively. On the contrary, *N*^5^-alkyl derivatives **2a** and **2b** and *N*^5^-2-acetamide derivatives **4c** and **4d** showed the opposite activity trend in comparison with compounds **3a**, **3b**, **4a**, **4b**, and **4e**, as they were more active against HCT116 than HepG2.

Compounds **4a** and **4b** were presented as the most promising amongst the tested compounds. Accordingly, they were further subjected to an evaluation of their cytotoxic effects on normal human cells. With determined IC_50_ values of 58.61 and 48.49 which translate into 100–14 selectivity indices, the results suggest that both compounds show good activity and selectivity towards cancerous cells rather than non-cancerous cells.

### 2.3. Structure Activity Relationship

**Figure 2:**
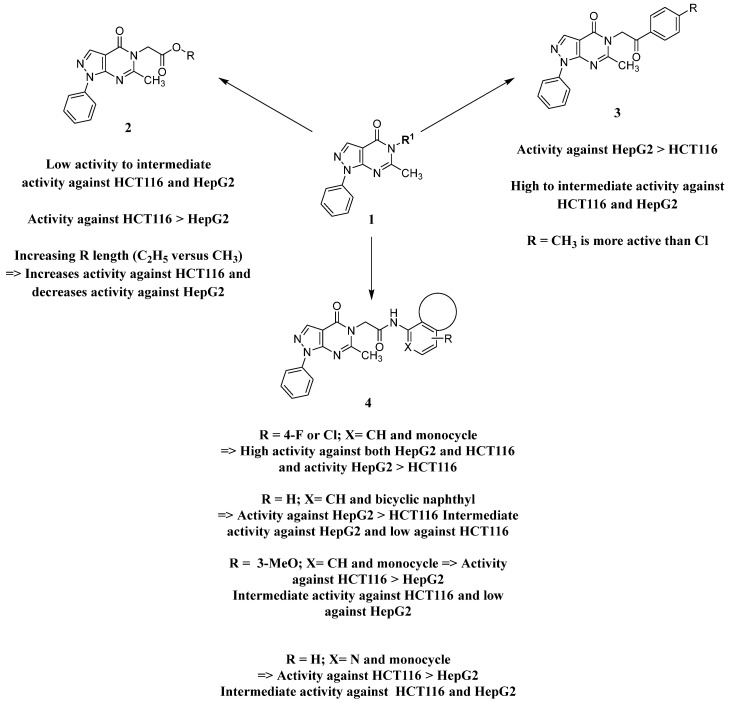
Structure–activity relationship analysis of *N^5^*-susbtituted derivatives.

Analysis of the structure-activity relationship of the evaluated compounds highlighted some possible key points (Figure 2). First, incorporation of an aromatic moiety within the introduced *N*^5^-subtituent was essential for bioactivity while aliphatic *N*^5^-subtituents free from aromatic moieties such as acetate ester derivatives **2** were not optimal for activity. The increase in alkyl ester length enhanced anti-HCT116 activity but was detrimental to anti-HepG2 activity. Such aliphatic derivatives were more active against HCT116 cells. Switching the *N*^5^-subtitution pattern to an aromatic moiety-containing phenacyl derivative **3** resulted in reversing the activity pattern where these compounds were more active against HepG2 than HCT116. Particularly, the *N*^5^-(4-methylphenacyl) derivative **3b** showed a potential IC_50_ of 1.90 µM against HepG2, which is seven-fold the potency of roscovitine. Increasing the distance of between the aromatic moiety and the *N*^5^-atom by one atom through the insertion of a nitrogen atom to convert phenacyl derivatives into *N*^5^-acetamide derivatives **4** resulted in mixed results depending on the aromatic moiety and the substitution pattern on it. When the aromatic moiety was a bicyclic unsubstituted 1-naphthyl moiety (compound **4e**), the activity pattern was maintained but the potency was not high. Not only was this activity pattern maintained upon using the monocyclic 4-halophenyl as the aromatic moiety in *N*^5^-acetamide derivatives **4a** and **4b**, highly active compounds were also obtained. Specifically, incorporation of the more electronegative 4-fluoro substituent (compound **4a**) afforded a more potent compound than the corresponding 4-chloro derivative (compound **4b**). Replacing the electronegative 4-halosusbtituent by the electron-donating 3-methoxy substituent (compound **4c**) resulted in lower activity and showed an activity trend opposite to compounds **4a** and **4b** in which compounds were more active against HCT116 than HepG2. Likewise, incorporation of the electron deficient pyridyl ring as the aromatic moiety showed the opposite activity trend in which compounds were more active against HCT116 than HepG2 but did not improve the activity. In summary, incorporation of the aromatic 4-susbituted-phenyl moiety in the *N*^5^-susbtituent resulted in activity enhancement affording the most active compounds.

### 2.4. CDK2 Inhibitory Activity

To confirm the inhibitory activity of the developed compounds on CDK2 as a mechanism of action involved in mediating cytotoxicity, the most effective cytotoxic compounds, **4a** and **4b**, were employed in in vitro enzymatic CDK2 inhibition assays [36]. The CDK2 inhibitor, roscovitine, was applied as the reference standard for comparison. As displayed in Table 2, the most potent cytotoxic compound tested, **4a**, showed a potent inhibition of CDK2 with a measured submicromolar IC_50_ of 0.21 µM, which is slightly better than the roscovitine reference. Meanwhile, the less effective cytotoxic compound **4b** elicited less potent inhibition of CDK2 with a determined IC_50_ value of 0.96 µM. Based on this outcome, it might be deduced that compounds **4a** and **4b** are potential CDK2 inhibitors and that their inhibition might be correlated with their observed antiproliferative activity.

### 2.5. In Silico Docking Study

**Figure 3:**
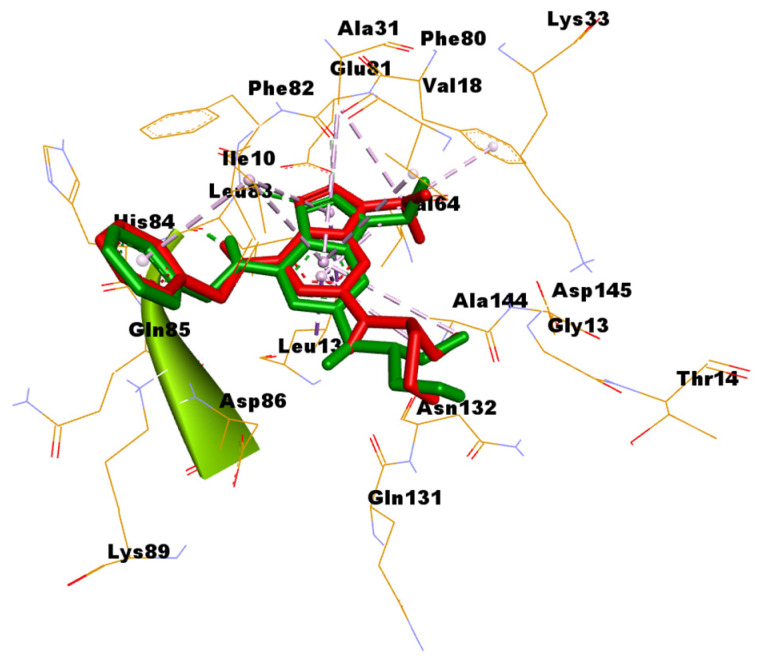
Superimposition of the original pose of roscovitine (green) and the redocked pose (red) into the substrate pocket of CDK2.

In silico docking was addressed to predict the binding mode between the most potent compounds **4a** and **4b** and the CDK2 enzyme. Among the numerous available crystal structures of CDK2, the X-ray crystal structure of CDK2 complexed with the roscovitine, the reference used in the conducted assay of CDK2 inhibition, was recruited for the docking simulation. Structurally, CDK2 has a typical folded bilobal architecture with a smaller *N*-terminal domain primarily consisting of β-sheets, while the larger *C*-terminal domain consists of α-helices. Orthostatic purine-analog inhibitors bind into a narrow cleft between the two lobal domains. The published literature has revealed three important main amino acids (Ile10, Leu83, and Leu134) for the binding inhibitor ligand [37,38]. The docking protocol was validated via redocking of roscovitine with its binding pocket of the enzyme. The root mean square deviation (RMSD) was 1.2942, suggesting that the docking protocol is reliable (Figure 3).

**Figure 4:**
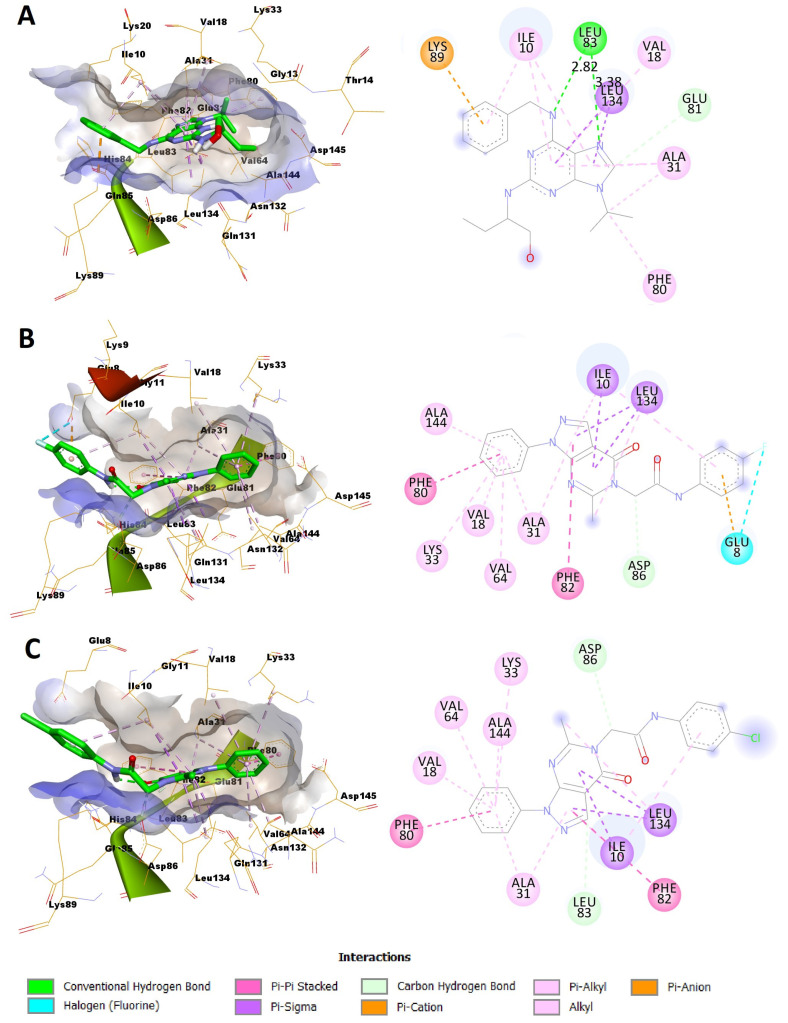
Predicted binding modes of the reference molecule roscovitine, and compounds **4a** and **4b** with CDK2: (**A**) predicted docking pose of roscovitine in the 3D (**right**) and 2D view (**left**) into the binding pocket of CDK2; (**B**) predicted docking pose of compound **4a** in the 3D (**right**) and 2D view (**left**) into the substrate pocket of CDK2; (**C**) predicted docking pose of compound **4b** in the 3D (**right**) and 2D view (**left**) into the substrate pocket of CDK2.

Exploration of the binding mode of roscovitine with its binding pocket unveiled that it could form various interactions with different amino acid residues within the ATP binding region of the enzyme including the three important amino acids (Ile10, Leu83, and Leu134) (Figure 4A, Table 3). It was capable of establishing two hydrogen bonds with the Leu83 amino acid residue via the N-atom of its purine ring system and NH of the benzyl amino side chain. The lengths of these two hydrogen bonds were 2.82 and 3.38 Å, respectively (Figure 4A, Table 3). Also, two π–sigma interactions were observed between Leu134 and the purine ring. The phenyl ring of the benzyl amino side chain formed a π–cation interaction with Lys89. In addition, various hydrophobic C–-H, π–alkyl, and alkyl interactions were established with amino acid residues within the binding site (Ile10, Val18, Ala31, Phe80, and Glu81). Compound **4a** was docked successfully into the ATP-binding site with a binding energy score of −7.0394 Kcal/mol (Figure 4B, Table 3), which was comparable to that of roscovitine which was −7.8236 Kcal/mol. It was able to establish a network of interactions including a halogen (fluorine) bond between its 4-fluoro substituent on the *N*-phenylacetamide moiety and Glu8. Moreover, the phenyl ring of the phenylacetamide side chain formed a π–anion interaction with Glu8. Furthermore, three π–sigma interactions were observed between Ile10 and Leu134, and the pyrazolo[3,4-*d*]pyrimidine ring. Additionally, various hydrophobic C–H, π–π stacked, π–alkyl, and alkyl interactions were established with amino acid residues within the binding site (Ile10, Val18, Ala31, Leu134, Ala144, Asp86, Lys33, Val64, Phe80, and Phe82). On the other hand, compound **4b** was docked to CDK2 with an acceptable binding energy score of −7.0358 Kcal/mol. Again, this docking score was comparable to that of the reference molecule, roscovitine. Interestingly, **4b** was able to establish the same interactions with the binding site as **4a** except the halogen and π–anion bonding with Glu8 (Figure 4C, Table 3). These results agreed with the biological and biochemical assays where compounds **4a** and **4b** were expected to dock successfully into the ATP-binding site of CDK2 and were able to interact with the essential amino acid residues within the binding site. Compound **4a** formed additional halogen and π–anion bonds with Glu8, illustrating that it might be beneficial to its binding process. This might be the reason why it possesses higher CDK2 inhibitory activity.

### 2.6. Predicted Physicochemical Properties and Toxicity Parameters

An oral bioavailability radar consisting of Lipinski’s rule of five and Veber’s rule was applied to obtain insights into the possible application of compounds **4a** and **4b** as oral anticancer agents [39,40]. Compliance of the tested molecules to these rules was investigated based on calculated molecular descriptors. Regarding the rules of Lipinski, violation of more than one parameter described in this rule indicates a high probability of oral bioavailability issues. In addition, Veber’s rule states that the calculated total polar surface area of a good oral drug should not exceed 140 and the number of rotatable bonds should not exceed 10. The OSIRIS software v2.16 was utilized for the calculation of the molecular parameters described in the tested rules. The calculated molecular descriptors of the investigated compounds suggested that both compounds would represent an acceptable orally administered molecule because no violations of the tested rules were anticipated (Table 4).

Moreover, OSIRIS was used to predict the in silico toxicity parameters of compounds **4a** and **4b**. Both compounds were predicted to possess no mutagenic, tumorigenic, or reproductive toxicities (Table 4). Collectively, the in silico predicted toxicity parameters suggested that the investigated compounds might be promising safe hits as anticancer agents.

## 3. Materials and Methods

### 3.1. Chemistry

Compounds were synthesized by adopting known synthetic procedures and were characterized as detailed in the Supplementary Information.

### 3.2. In Vitro Assay of Antiproliferative Activity

In vitro evaluation of growth inhibition assay was conducted according to the well-known MTT assay protocol [33,34] as detailed in the Supplementary Information.

### 3.3. In Vitro Assay of CDK2 Inhibition

In vitro evaluation of CDK2 inhibition was determined using a commercially available assay kit from BPS Bioscience Inc. (San Diego, CA, USA) as described in the Supplementary Information [41].

### 3.4. Statistical Analysis

Results are the mean of triplicate experiments. Significance was confirmed relative to control at *p* values ≤ 0.05 via one-way ANOVA.

### 3.5. In Silico Docking Study

The docking study was performed using the crystal structure of human CDK2 co-crystallized with roscovitine (PDB ID: 2A4L) [37] according to known standard protocols. Autodock was utilized and results were visualized utilizing the DS visualizer [42,43,44]. Ligands’ structures were sketched and prepared by energy minimization. The active site was identified as the site where the original co-crystallized inhibitor (roscovitine) existed. Validation of the docking protocol was performed via redocking of roscovitine with the crystal structure of the enzyme. The original conformation was reproduced and the RMSD value was 1.2942.

## 4. Conclusions

New pyrazolo[3,4-*d*]pyrimidine analogs were designed as CDK2 inhibitors aimed at obtaining new potent hits as anticancer molecules. The designed target compounds were synthesized and evaluated for their cytotoxic activity against two cell lines, viz., HCT116 and HepG2 cells, which are CRC and HCC cell lines, respectively. Compounds **4a** and 4b exhibited superior activity relative to a reference standard against the tested cell lines. Exploration of their CDK2 inhibitory activities showed correlations with their observed antiproliferative potencies and confirmed them as CDK2 inhibitors. Compound **4a** was identified as the most potent amongst the tested compounds (with IC_50_ value of 0.21 µM) showing superior antiproliferative activity and CDK2 inhibition in comparison with the reference standard, roscovitine (with IC_50_ value of 0.25 µM). Molecular docking provided insights supporting the observed in vitro results. Each of the compounds **4a** and **4b** successfully docked into the CDK2 ATP-binding site, establishing a network of interactions with essential amino acid residues within the active site. The 4-fluoro substituent in compound **4a** resulted in additional hydrogen and halogen bonds explaining the higher potency of compound **4a**. Together, these results introduce compounds **4a** and **4b** as interesting new hits endowed with potential CDK2 inhibition and antiproliferative activity against CRC and HCC cell lines, HCT116 and HepG2. These two compounds (**4a** and **4b**) might serve as potential anticancer hits for further development and evaluation of their in vivo activity. Also, their possible route of administration should be investigated.

## Figures and Tables

**Table 1:** IC_50_ values of synthesized compounds and standard references against HCT116, HepG2, and WI-38 cell lines.

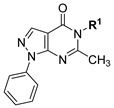				
Compound	R^1^	IC_50_ ± SD (µM) ^a^		
		HCT116	HepG2	WI-38
**2a**	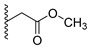	19.40 ± 0.86	32.89 ± 1.67	NT ^b^
**2b**	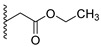	10.91 ± 0.48	58.00 ± 2.94	NT ^b^
**3a**	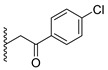	35.85 ± 1.59	26.25 ± 1.33	NT ^b^
**3b**	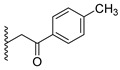	10.75 ± 0.48	1.90 ± 0.10	NT ^b^
**4a**	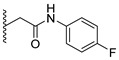	1.09 ± 0.05	0.58 ± 0.03	58.61
**4b**	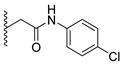	3.38 ± 0.15	2.57 ± 0.13	48.49
**4c**	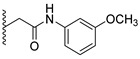	26.35 ± 1.17	87.27 ± 4.42	NT ^b^
**4d**	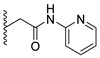	27.04 ± 1.20	41.60 ± 2.11	NT ^b^
**4e**	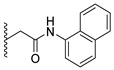	41.23 ± 1.83	16.08 ± 0.81	NT ^b^
**Roscovitine**	—	12.24 ± 1.17	13.82 ± 1.15	17.31

^a^ IC_50_ values were calculated from triplicate experiments. ^b^ NT: compounds were not tested.

**Table 2:** Results of in vitro CDK2 inhibition assay of compounds **4a** and **4b** (IC_50_ values in µM).

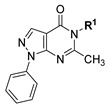		
Compound	R^1^	CDK2 Inhibition IC_50_ ± SD (µM) ^a^
**4a**	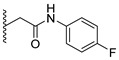	0.21 ± 0.01
**4b**	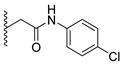	0.96 ± 0.05
**Roscovitine**	—	0.25 ± 0.01

^a^ IC_50_ values were calculated from triplicate experiments.

**Table 3:** Calculated binding energy scores of compounds **4a** and **4b** and type of binding interactions of the target compounds (**4a** and **4b**) with the crystal structure of CDK2.

Comp.	Binding Energy Score ^a^	Type of Binding Interactions
**4a**	−7.0394	One halogen (fluorine) bond between its 4-fluoro substituent on the *N*-phenylacetamide moiety and Glu8. One π–anion interaction between the phenyl ring of the phenylacetamide side chain and Glu8. Three π–sigma interactions between Ile10 and Leu134, and the pyrazolo[3,4-*d*]pyrimidine ring. Various hydrophobic C–H, π–π stacked, π–alkyl, and alkyl interactions with amino acid residues within the binding site (Ile10, Val18, Ala31, Leu134, Ala144, Asp86, Lys33, Val64, Phe80, and Phe82).
**4b**	−7.0358	Three π–sigma interactions between Ile10 and Leu134, and the pyrazolo[3,4-d]pyrimidine ring. Various hydrophobic C–H, π–π stacked, π–alkyl, and alkyl interactions with amino acid residues within the binding site (Ile10, Val18, Ala31, Leu134, Ala144, Asp86, Lys33, Val64, Phe80, and Phe82).
**Roscovitine**	−7.8236	Two hydrogen bonds with the Leu83 amino acid residue via the N-atom of its purine ring system and NH of the benzyl amino side chain. Two π–sigma interactions between Leu134 and the purine ring. One π–cation interaction between the phenyl ring of the benzyl amino side chain and Lys89. Various hydrophobic C–H, π–alkyl, and alkyl interactions with amino acid residues within the binding site (Ile10, Val18, Ala31, Phe80, and Glu81).

^a^ Binding energy score in Kcal/mol.

**Table 4:** Calculated molecular descriptors and predicted toxicity parameters of compounds **4a** and **4b**.

Comp.	Lipinski’s Rule				Veber’s Rule		nVs ^g^	Predicted Toxicity Parameters		
	cLogP ^a^	M.Wt. ^b^	nHBA ^c^	nHBD ^d^	nRB ^e^	TPSA ^f^		Mutagenic	Tumorigenic	Reproductive
**4a**	1.4553	377.378	7	1	4	79.59	0	none	none	none
**4b**	1.9605	393.833	7	1	4	79.59	0	none	none	none

^a^ Calculated partition coefficient, ^b^ molecular weight, ^c^ number of hydrogen bond acceptors, ^d^ number of hydrogen bond donors, ^e^ number of rotatable bonds, ^f^ total polar surface area, ^g^ number of violations to the rules.

## Data Availability

Data are contained within the article or Supplementary Material.

## Supplementary Materials

The following supporting information can be downloaded at: https://www.mdpi.com/article/10.3390/ph16111593/s1, Experimental procedures and NMR spectra.

## Abbreviations

HCC	Hepatocellular carcinoma
CRC	Colorectal cancer
IARC	International Agency for Research on Cancer
CDK2	Cyclin-dependent kinase-2
SD	Standard deviation
IC_50_	Half-maximal inhibitory concentration
RMSD	Root mean square deviation
