# Supplementary material for: Design, Synthesis, In Vitro, and In Silico Studies of New N5-Substituted-pyrazolo[3,4-d]pyrimidinone Derivatives as Anticancer CDK2 Inhibitors
Source: Pharmaceuticals (Basel). 2023 Nov 11;16(11):1593. doi: 10.3390/ph16111593 (PMC10674233; doi:10.3390/ph16111593)
Supplement: Supplementary file 1 [file pharmaceuticals-16-01593-s001.zip › pharmaceuticals-2674660-supplementary.pdf]

Supplementary Information for

Design, Synthesis, In Vitro, and In Silico Studies of New N^5-Substituted-pyrazolo[3,4-*d*]pyrimidinone Derivatives as Anticancer CDK2 Inhibitors

Waheed A. Zaki [1,†], Selwan M. El-Sayed [2,†], Mohamed Alswah [1,*], Ahmed El-Morsy [1,3], Ashraf H. Bayoumi [1], Abrahman S. Mayhoub [1,4], Walaa H. Moustafa [5], Aeshah A. Awaji [6], Eun Joo Roh [7,8,*], Ahmed H.E. Hassan [2] and Kazem Mahmoud [9]

[1] Department of Pharmaceutical Organic Chemistry, Faculty of Pharmacy, Al-Azhar University, Cairo 11884, Egypt
[2] Department of Medicinal Chemistry, Faculty of Pharmacy, Mansoura University, Mansoura 35516, Egypt
[3] Pharmaceutical Chemistry Department, College of Pharmacy, The Islamic University, Najaf 54001, Iraq
[4] Nanoscience Program, University of Science and Technology, Zewail City of Science and Technology, October Gardens, 6th of October, Giza 12578, Egypt
[5] Microbiology and Immunology Department, Faculty of Pharmacy, Helwan University, Cairo 19448, Egypt
[6] Department of Biology, Faculty of Science, University College of Taymaa, University of Tabuk, Tabuk 71491, Saudi Arabia
[7] Chemical and Biological Integrative Research Center, Korea Institute of Science and Technology (KIST), Seoul 02792, Republic of Korea
[8] Division of Bio-Medical Science & Technology, University of Science and Technology, Daejeon 34113, Republic of Korea
[9] Department of Pharmaceutical Chemistry, Faculty of Pharmacy, Egyptian Russian University, Badr City 11829, Egypt
* Correspondence: drmohammedalswah@azhar.edu.eg (M.A.); r8636@kist.re.kr (E.J.R.)
† These authors contributed equally to this work.

1. Chemistry

General Procedure for synthesis of *N*^{5}-alkylated--6-methyl-1-phenyl-1,5-dihydro-4*H*-pyrazolo[3,4-*d*]pyrimidin-4-ones (2–4)

A mixture of 6-methyl-1,5-dihydro-1-phenyl-4*H*-pyrazolo[3,4-*d*]pyrimidin-4-one (**1**, 0.01 mol), the appropriate alkyl halide derivative (0.01 mol) and anhydrous potassium carbonate (0.01 mol) in DMF (20 ml) was heated on a water bath for 5-24 hrs. The reaction mixture was poured onto crushed ice with continuous stirring. The obtained solid was filtered and crystallized using the proper solvent system.

Methyl 2-(6-methyl-4-oxo-1-phenyl-1,5-dihydro-4*H*-pyrazolo[3,4-*d*]pyrimidin-5-yl)acetate (2a)

Crystallized from ethanol as white crystals. Yield = 75%; m.p. 140-141 °C; ^{1}H-NMR (DMSO-*d*$_6$) δ 8.36 (s, 1H), 8.06 (d, J = 7.99 Hz, 2H), 7.59 (t, J = 7.82 Hz, 2H), 7.43 (t, J = 7.24 Hz, 1H), 4.98 (s, 2H), 3.75 (s, 3H), 2.60 (s, 3H); ^{13}C-NMR (DMSO-*d*$_6$) δ 168.96, 159.79, 157.47, 150.83, 138.59, 136.61, 129.75 (2C), 127.65, 122.23 (2C), 105.09, 53.06, 45.54, 23.98; HRMS: *m*/*z* found 299.1149 (Calc. for $C_{15}H_{15}N_4O_3^+$ [M+H]$^+$ 299.1139).

Ethyl 2-(4-oxo-6-methyl-1-phenyl-1,5-dihydro-4*H*-pyrazolo[3,4-*d*]pyrimidin-5-yl)acetate (2b)

Crystallized from ethanol as white crystals. Yield = 75%; m.p. 138-140°C; ^{1}H-NMR (DMSO-*d*$_6$) δ 8.36 (s, 1H), 8.06 (d, , J = 7.64 Hz, 2H), 7.58 (t, , J = 7.90 Hz, 2H), 7.42 (t, , J = 7.35 Hz, 1H), 4.97 (s, 2H), 4.21 (q, , J = 7.06 Hz, 2H), 2.60 (s, 3H), 1.25 (t, , J = 7.08 Hz, 3H); ^{13}C-NMR (DMSO-*d*$_6$) δ 168.47, 159.78, 157.46, 150.83, 138.60, 136.61, 129.73 (2C), 127.62, 122.19 (2C), 105.09, 61.99, 45.59, 23.97, 14.46; HRMS: *m*/*z* found 313.1304 (Calc. for $C_{16}H_{17}N_4O_3^+$ [M+H]$^+$ 313.1295).

5-(2-(4-Chlorophenyl)-2-oxoethyl)-6-methyl-1-phenyl-1,5-dihydro-4*H*-pyrazolo[3,4-*d*]pyrimidin-4-one (3a)

Crystallized from ethanol as buff crystals. Yield = 65 %; m.p. 178-180°C; ^{1}H-NMR (DMSO-d_6) δ 8.36 (s, 1H), 8.17 (d, J = 9.31 Hz, 2H), 8.10 (d, J = 8.01 Hz, 2H), 7.73 (d, J = 8.51 Hz, 2H), 7.60 (t, J = 7.72 Hz, 2H), 7.44 (t, J = 7.46 Hz, 1H), 5.79 (s, 2H), 2.55 (s, 3H); ^{13}C-NMR (DMSO-d_6) δ 192.80, 160.07, 157.48, 151.01, 139.74, 138.67, 136.61, 133.46, 130.70 (2C), 129.77 (2C), 129.63 (2C), 127.61, 122.21 (2C), 105.17, 50.85, 24.07; HRMS: *m*/*z* found 417.0849 (Calc. for $C_{20}H_{15}ClKN_4O_2^+$ [M+H]$^+$ 417.0515).

5-(2-(4-Methylphenyl)-2-oxoethyl)-6-methyl-1-phenyl-1,5-dihydro-4*H*-pyrazolo[3,4-*d*]pyrimidin-4-one (3b)

Crystallized from ethanol as white crystals. Yield = 62%; m.p. 185-186°C; ^{1}H-NMR (DMSO-d_6) δ 8.36 (s, 1H), 8.10 (d, J = 8.19 Hz, 2H), 8.05 (d, J = 8.31 Hz, 2H), 7.63–7.57 (m, 4H), 7.45 (d, J = 8.07 Hz, 2H), 7.41 (t, J = 7.21 Hz, 1H), 5.77 (s, 2H), 2.54 (s, 3H), 2.45 (s, 3H); ^{13}C-NMR (DMSO-d_6) δ 193.05, 160.10, 157.52, 151.01, 138.69, 136.61, 132.31, 130.02 (2C), 129.77 (2C), 129.67, 128.84 (2C), 127.60, 122.21 (2C), 105.19, 50.65, 24.03, 21.79; HRMS: *m*/*z* found 359.15163 (Calc. for $C_{21}H_{19}N_4O_2^+$ [M+H]$^+$ 359.1503).

***N*-(4-Fluorophenyl)-2-(6-methyl-4-oxo-1-phenyl-1,5-dihydro-4*H*-pyrazolo[3,4-*d*]pyrimidin-5-yl)acetamide (4a)**

Crystallized from ethanol as brown crystals. Yield = 60%; m.p. 259-260°C; ^{1}H-NMR (DMSO-d_6) δ 10.56 (brs, 1H), 8.35 (s, 1H), 8.09 (d, J = 8.01 Hz, 2H), 7.64–7.56 (m, 4H), 7.43 (t, J = 7.41 Hz, 1H), 7.14 (t, J = 8.70 Hz, 2H), 5.00 (s, 2H), 2.63 (s, 3H); ^{13}C-NMR (DMSO-d_6) δ 165.75, 160.39, 157.69, 150.96, 138.70, 136.60, 135.43 ($J_{C\text{-}F}$ = 2.51 Hz), 129.75 (2C), 127.55, 122.11

(2C), 121.42 ($J_{C\text{-}F}$ = 7.82 Hz, 2C), 115.95 ($J_{C\text{-}F}$ = 22.17 Hz, 2C), 105.26, 47.00, 24.20; HRMS: *m*/*z* found 378.1373 (Calc. for $C_{20}H_{17}FN_5O_2^+$ $[M+H]^+$ 378.1361).

_N_-(4-Chlorophenyl)-2-(6-methyl-4-oxo-1-phenyl-1,5-dihydro-4_H_-pyrazolo[3,4-_d_]pyrimidin-5-yl)acetamide (4b)

Crystallized from ethanol as brown crystals. Yield = 60 %; m.p. 220-221°C; ^{1}H-NMR (DMSO-*d*$_6$) δ 10.64 (brs, 1H), 8.36 (s, 1H), 8.09 (d, J = 8.00 Hz, 2H), 7.66–7.56 (m, 4H), 7.46–7.37 (m, 3H), 5.01 (s, 2H), 2.63 (s, 3H); ^{13}C-NMR (DMSO-*d*$_6$) δ 166.02, 160.40, 157.68, 150.96, 138.69, 137.99, 136.61, 129.78 (2C), 129.29 (2C), 127.68, 127.58, 122.14 (2C), 121.21 (2C), 105.24, 47.13, 24.22; HRMS: *m*/*z* found 394.1076 (Calc. for $C_{20}H_{17}ClN_5O_2^+$ $[M+H]^+$ 394.1065).

_N_-(3-Methoxyphenyl)-2-(6-methyl-4-oxo-1-phenyl-1,5-dihydro-4_H_-pyrazolo[3,4-_d_]pyrimidin-5-yl)acetamide (4c)

Crystallized from ethanol as brown needles. Yield = 62%; m.p. 220-225°C; ^{1}H-NMR (DMSO-*d*$_6$) δ 10.51 (s, 1H), 8.35 (s, 1H), 8.11–8.05 (m, 2H), 7.60–7.55 (m, 2H), 7.43 (t, J = 7.72 Hz, 1H), 7.33 (s, 1H), 7.25 (t, J = 8.18 Hz, 1H) 7.13 (d, J = 8.08 Hz, 1H), 6.67 (d, J = 8.23 Hz, 1H), 5.01 (s, 2H), 3.73 (s, 3H), 2.62 (s, 3H); ^{13}C-NMR (DMSO-*d*$_6$) δ 165.85, 160.38, 159.07, 157.69, 150.96, 140.23, 138.71, 136.59, 130.17, 129.75 (2C), 127.54, 122.11 (2C), 111.84, 109.65, 105.33, 55.45, 47.09, 24.20; HRMS: *m*/*z* found 390.1577 (Calc. for $C_{21}H_{20}N_5O_3^+$ $[M+H]^+$ 390.1561).

2-(6-Methyl-4-oxo-1-phenyl-1,5-dihydro-4_H_-pyrazolo[3,4-_d_]pyrimidin-5-yl)-_N_-(pyridin-3-yl)acetamide (4d)

Crystallized from ethanol as orange needles. Yield = 65%; m.p. 222-223°C; ^{1}H-NMR (DMSO-*d*$_6$) δ 11.26 (s, 1H), 8.43 (s, 1H), 8.35 (s, 1H), 8.12–8.02 (m, 3H), 7.93 (dd, J_1 = 8.88 Hz, J_2 =

2.64 Hz, 2H), 7.60 (t, *J* = 7.75 Hz, 2H), 7.43 (t, *J* = 7.39 Hz, 1H), 5.09 (s, 2H), 2.61 (s, 3H); ^{13}C-NMR (DMSO-*d*$_6$) *δ* 166.97, 160.37, 157.66, 150.96, 150.68, 138.69, 138.60, 136.61, 129.76 (2C), 127.57, 125.94, 122.13 (2C), 115.14, 105.23, 47.20, 24.18.

***N*-(Naphthalen-1-yl)-2-(6-methyl-4-oxo-1-phenyl-1,5-dihydro-4*H*-pyrazolo[3,4-*d*]pyrimidin-5-yl)acetamide (4e)**

Crystallized from ethanol as green needles. Yield = 65%; m.p. 243-244°C; ^{1}H-NMR (DMSO-*d*$_6$) *δ* 10.47 (brs, 1H), 8.39 (s, 1H), 8.19 (d, *J* = 8.21 Hz, 1H), 8.10 (d, *J* = 7.58 Hz, 1H), 7.98 (d, *J* = 7.68 Hz, 1H), 7.82 (d, *J* = 8.16 Hz, 1H), 7.71 (d, *J* = 7.37 Hz, 2H), 7.63-7.49 (m, 4H), 7.43 (t, *J* = 7.41 Hz, 1H), 5.19 (s, 2H), 2.41 (s, 3H); ^{13}C-NMR (DMSO-*d*$_6$) *δ* 166.80, 160.41, 157.80, 151.01, 138.72, 136.65, 134.20, 133.38, 129.77 (2C), 128.66, 128.24, 127.55, 126.65, 126.51, 126.19, 126.07, 123.19, 122.12 (2C), 105.35, 47.00, 24.27; HRMS: *m*/*z* found 432.1444 (Calc. for C24H19N5NaO2+ $[M+H]^+$ 432.1431).

2. Biological evaluations

2.1. *In vitro* antiproliferative activity

Solutions of MTT (3-[4,5-dimethylthiazol-2-yl]-2,5-diphenyl tetrazolium bromide), dissolved in medium or balanced salt solutions without phenol red, are yellowish in color. Mitochondrial dehydrogenases of viable cells cleave the tetrazolium ring, yielding purple formazan crystals which are insoluble in aqueous solutions. The crystals are dissolved in acidified isopropanol. The resulting purple solution is spectrophotometrically measured. An increase or decrease in cell number results in a concomitant change in the amount of formazan formed, indicating the degree of cytotoxicity caused by the test material. Firstly, cultures were removed from incubator into laminar flow hood or other sterile work area. Then, reconstitution of each vial of MTT [M-5655] was done to be used with 3 ml of medium or balanced salt solution without phenol red and

serum. After that, the reconstituted MTT was added in an amount equal to 10% of the culture medium volume. Cultures were returned to incubator for 2-4 hours depending on cell type and maximum cell density. Incubation times should be consistent when making comparisons. After the incubation period, cultures were removed from incubator and the resulting formazan crystals were dissolved by adding an amount of MTT Solubilization Solution [M-8910] equal to the original culture medium volume. Occasionally, especially in dense cultures, pipetting up and down [trituration] may be required to completely dissolve the MTT formazan crystals. Absorbance was measured spectrophotometrically at a wavelength of 570 nm. The background absorbance of multiwell plates was measured at 690 nm and subtract from the 570 nm measurement.

2.2. CDK2/Cyclin A2 inhibitory activity

Promega Kinase-Glo Plus luminescence kinase assay protocol was adopted as reported [1]. All samples and controls should be tested in duplicate. Thaw 5x Kinase assay buffer 1, ATP and 10x CDK substrate peptide 1. Prepare the master mixture (25 µl per well): N wells x (6 µl 5x Kinase assay buffer 1 + 1 µl ATP (500 µM) + 5 µl 10x CDK substrate peptide 1 + 13 µl distilled water). Add 25 µl to every well. Add 5 µl of Inhibitor solution of each well labeled as “Test Inhibitor”. For the “Positive Control" and “Blank”, add 5 µl of the same solution without inhibitor (Inhibitor buffer). Prepare 3 ml of 1x Kinase assay buffer 1 by mixing 600 µl of 5x Kinase assay buffer 1 with 2400 µl water. 3 ml of 1x Kinase assay buffer 1 is sufficient for 100 reactions. To the wells designated as "Blank", add 20 µl of 1x Kinase assay buffer 1. Thaw CDK2/CyclinA2 enzyme on ice. Upon first thaw, briefly spin tube containing enzyme to recover full content of the tube. Calculate the amount of CDK2/CyclinA2 required for the assay and dilute enzyme to ~5 ng/µl with 1x Kinase assay buffer 1. Store remaining undiluted enzyme in aliquots at -80°C. Initiate

reaction by adding 20 μl of diluted CDK2/CyclinA2 enzyme to the wells designated "Positive Control" and "Test Inhibitor Control". Incubate at 30°C for 45 minutes. Thaw Kinase-Glo Max reagent. After the 45-minute reaction, add 50 μl of Kinase-Glo Max reagent to each well. Cover plate with aluminum foil and incubate the plate at room temperature for 15 minutes. Measure luminescence using the microplate reader. "Blank" value is subtracted from all readings.

References:

[1] J. Hennek, J. Alves, E. Yao, S.A. Goueli, H. Zegzouti, Bioluminescent kinase strips: A novel approach to targeted and flexible kinase inhibitor profiling, Anal. Biochem., 495 (2016) 9.

NMR Spectra

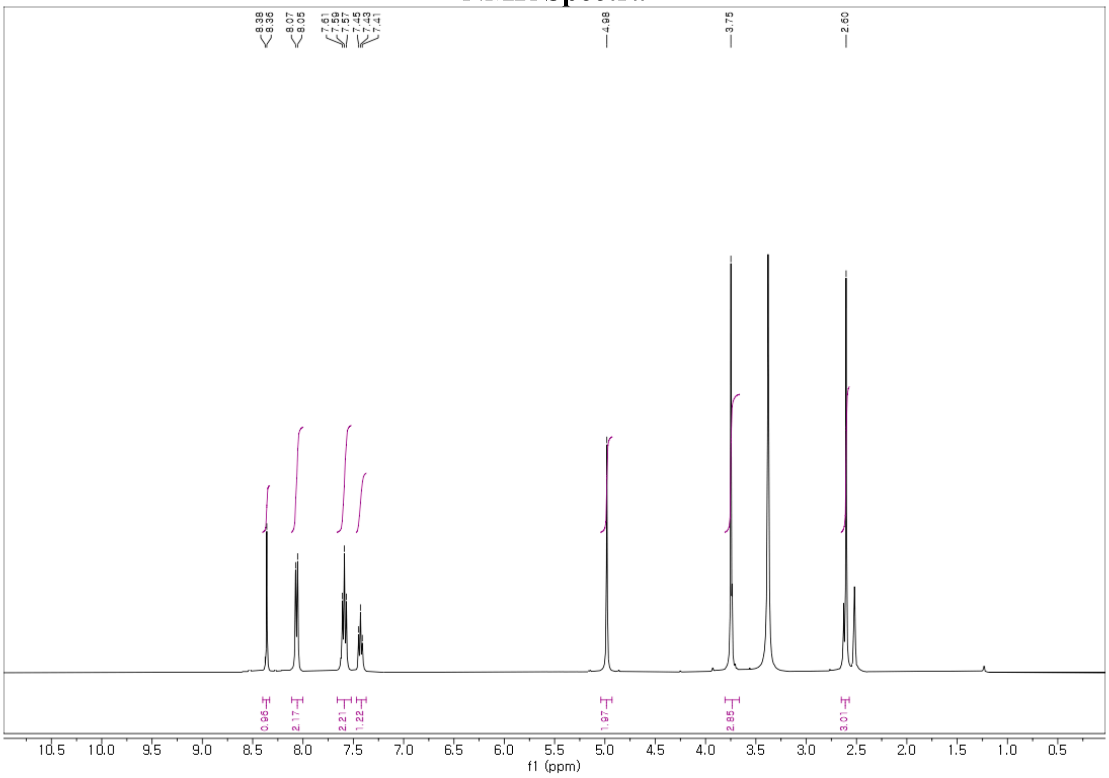

^{1}H-NMR spectra of compound 2a in DMSO-d_6

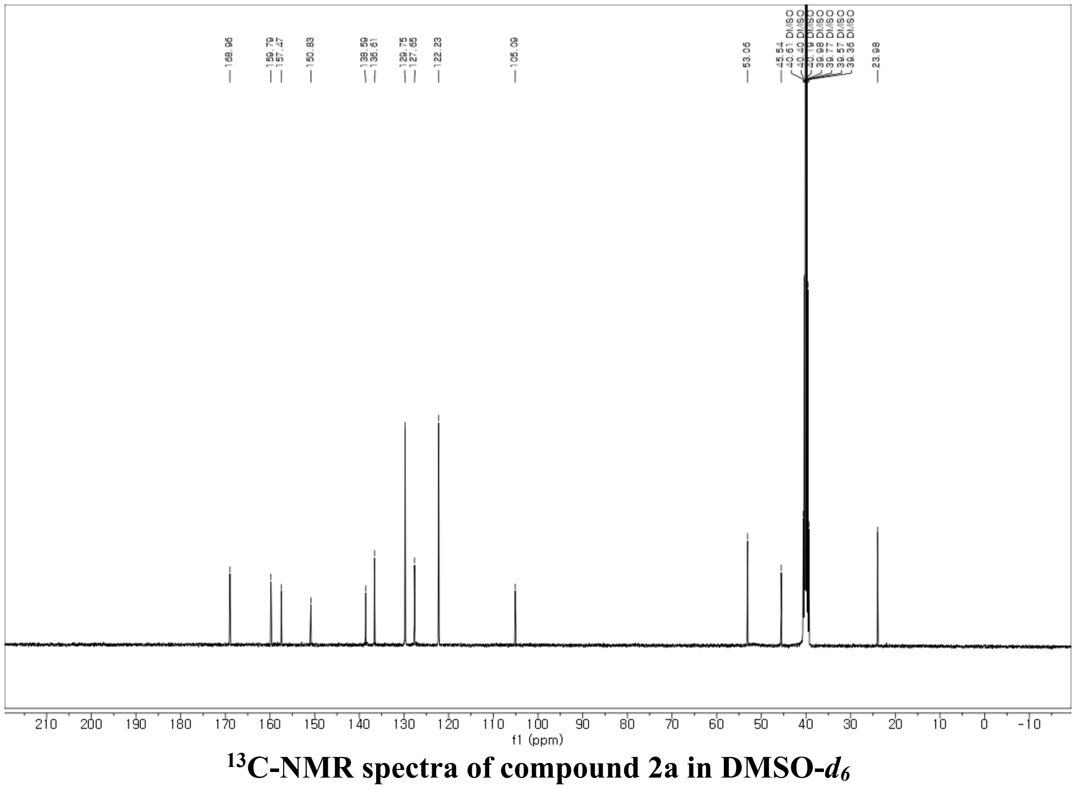

^{13}C-NMR spectra of compound 2a in DMSO-d_6

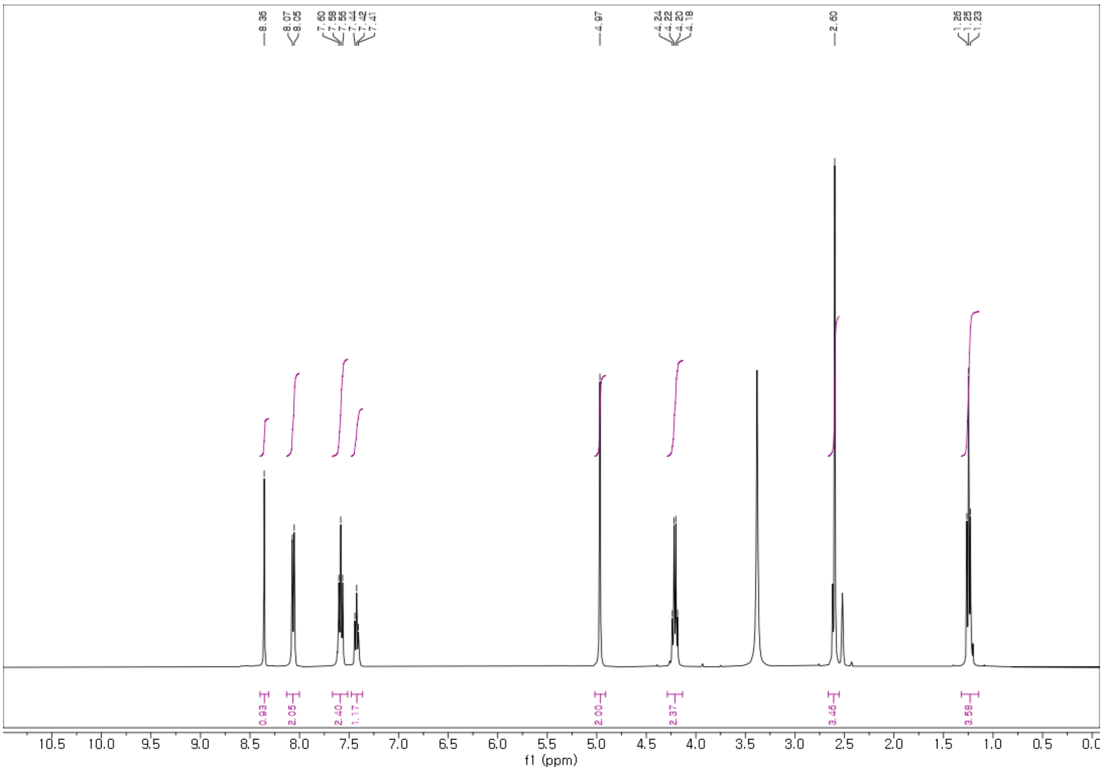

^{1}**H-NMR spectra of compound 2b in DMSO-*d*$_6$**

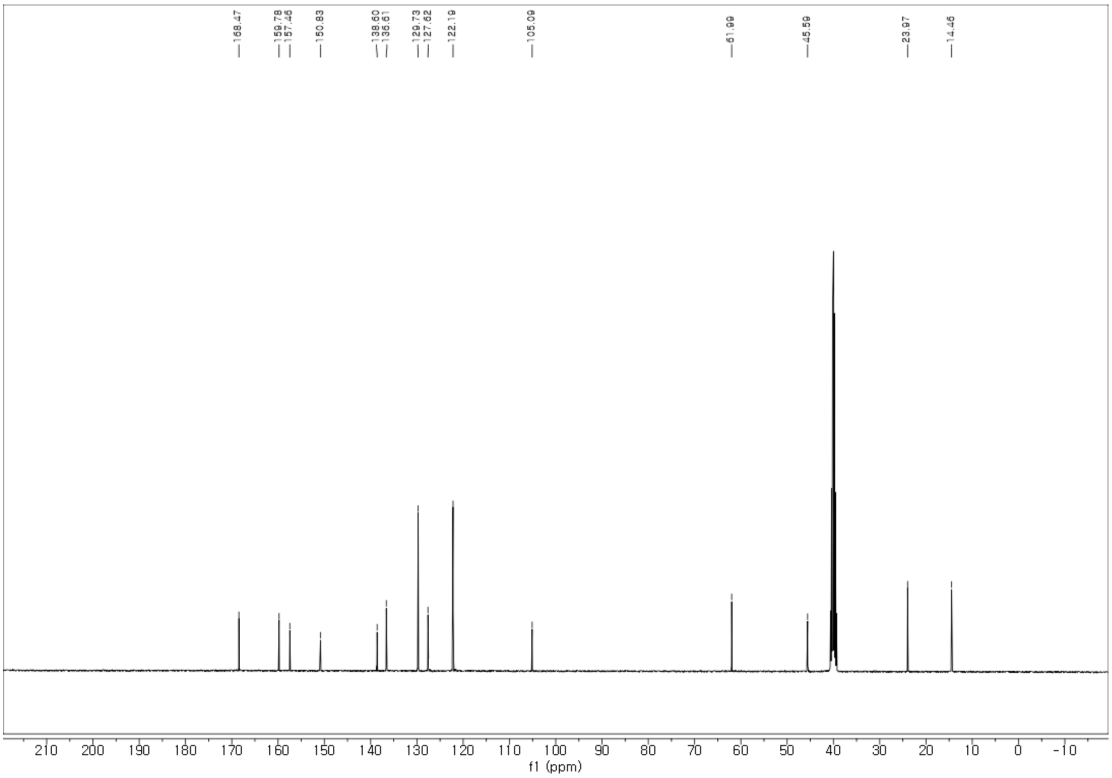

^{13}**C-NMR spectra of compound 2b in DMSO-*d*$_6$**

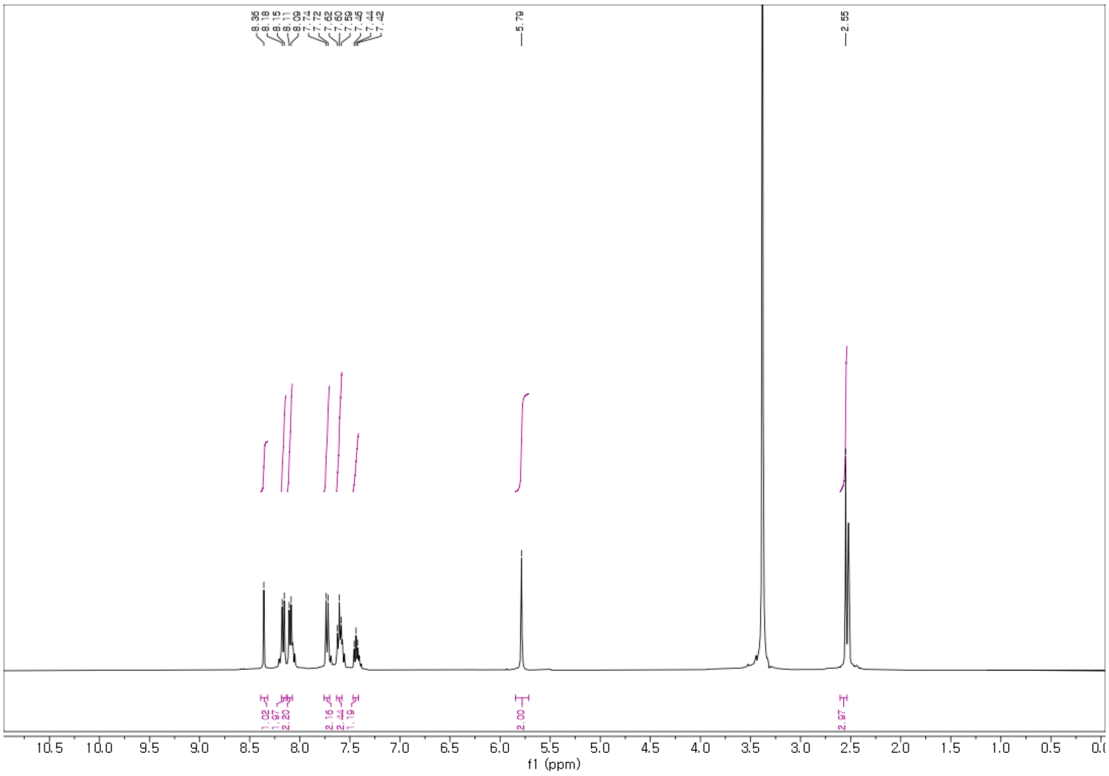

^{1}H-NMR spectra of compound 3a in DMSO-*d₆*

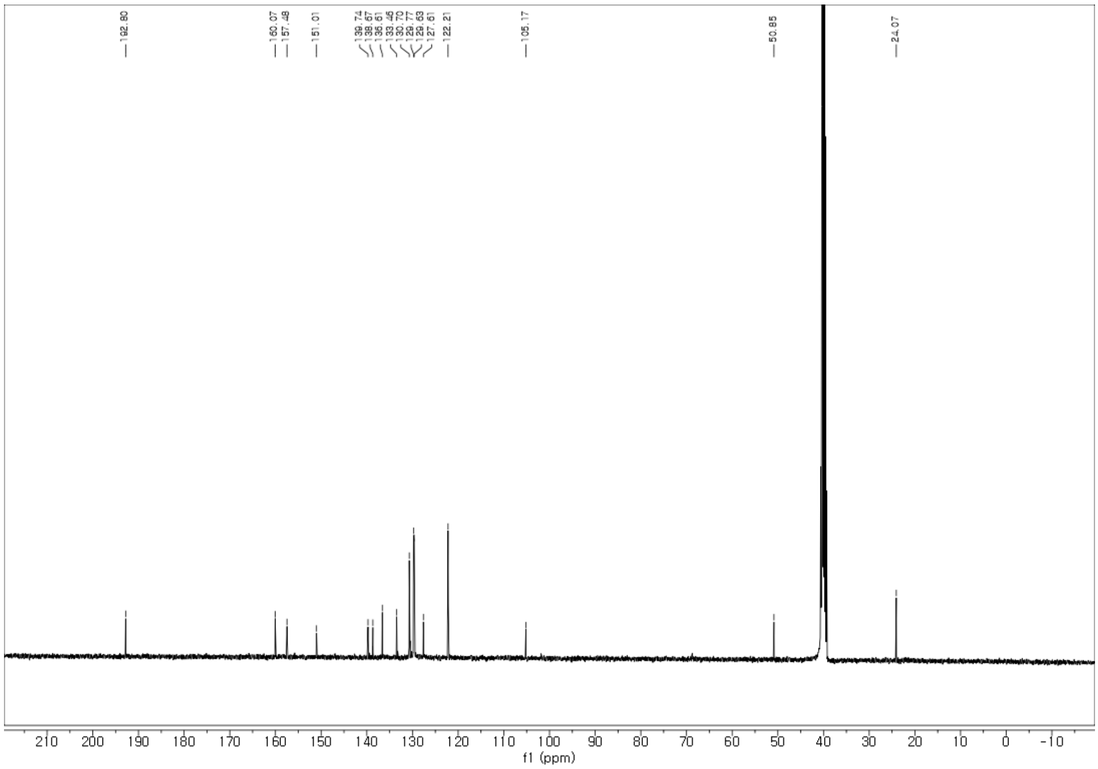

^{13}C-NMR spectra of compound 3a in DMSO-*d₆*

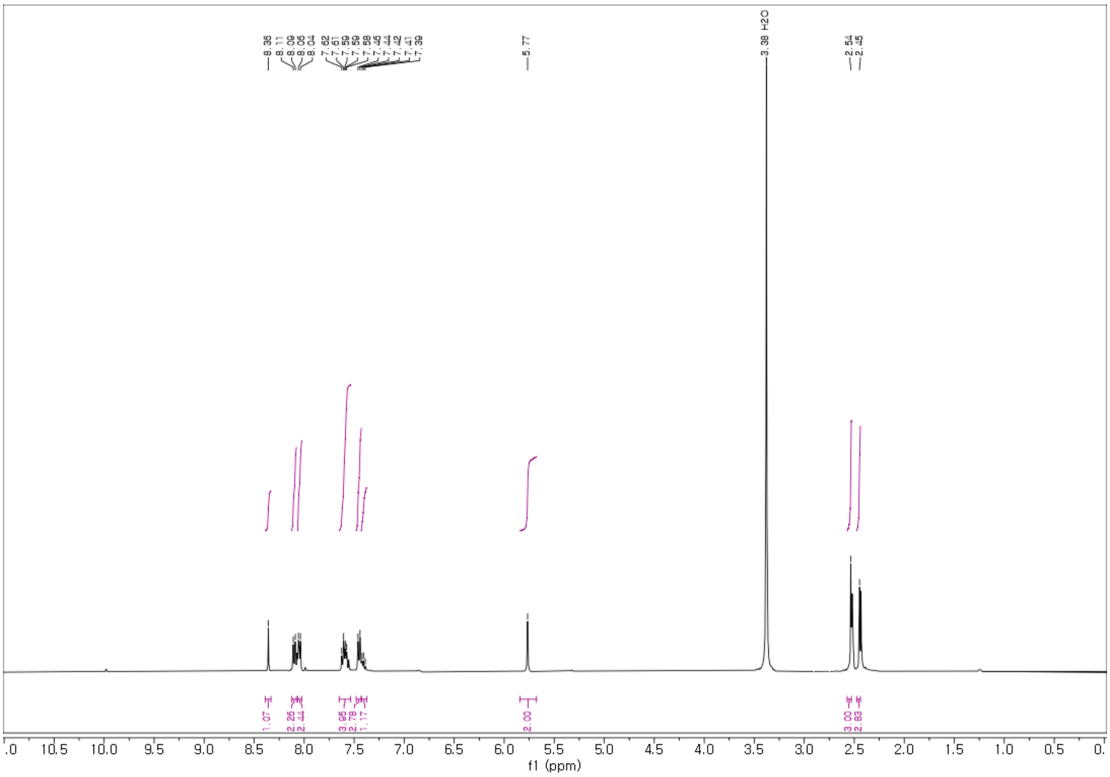

^{1}H-NMR spectra of compound 3b in DMSO-*d_6*

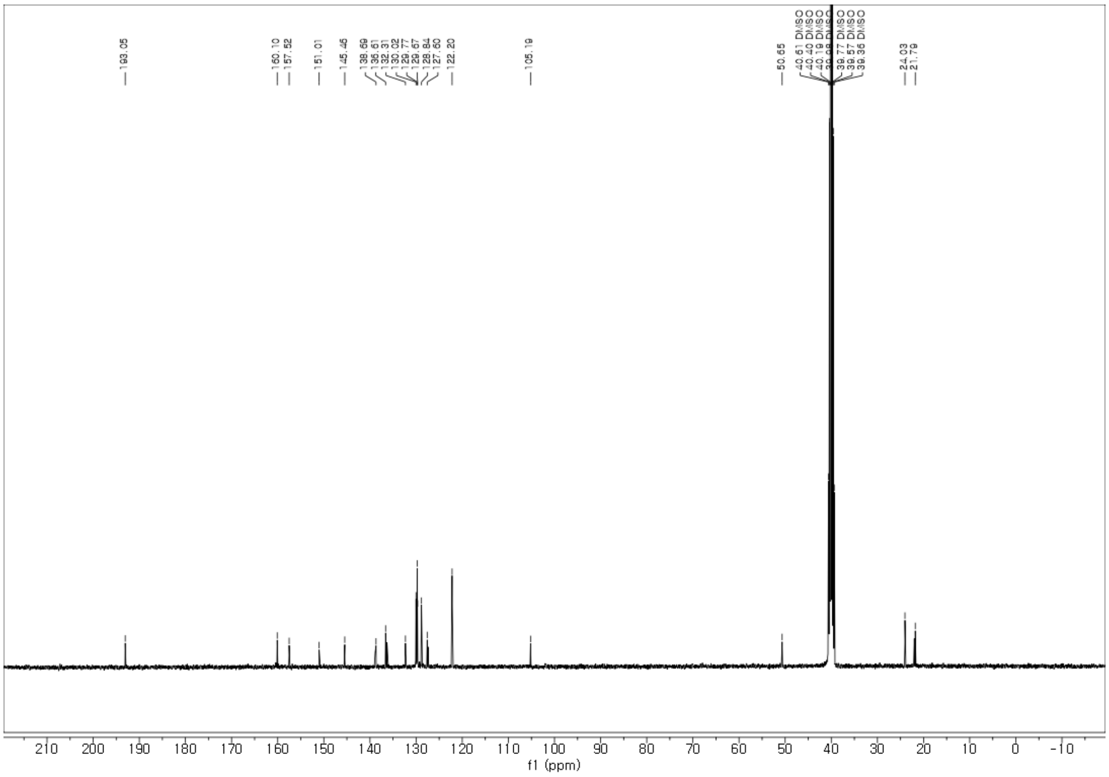

^{13}C-NMR spectra of compound 3b in DMSO-*d_6*

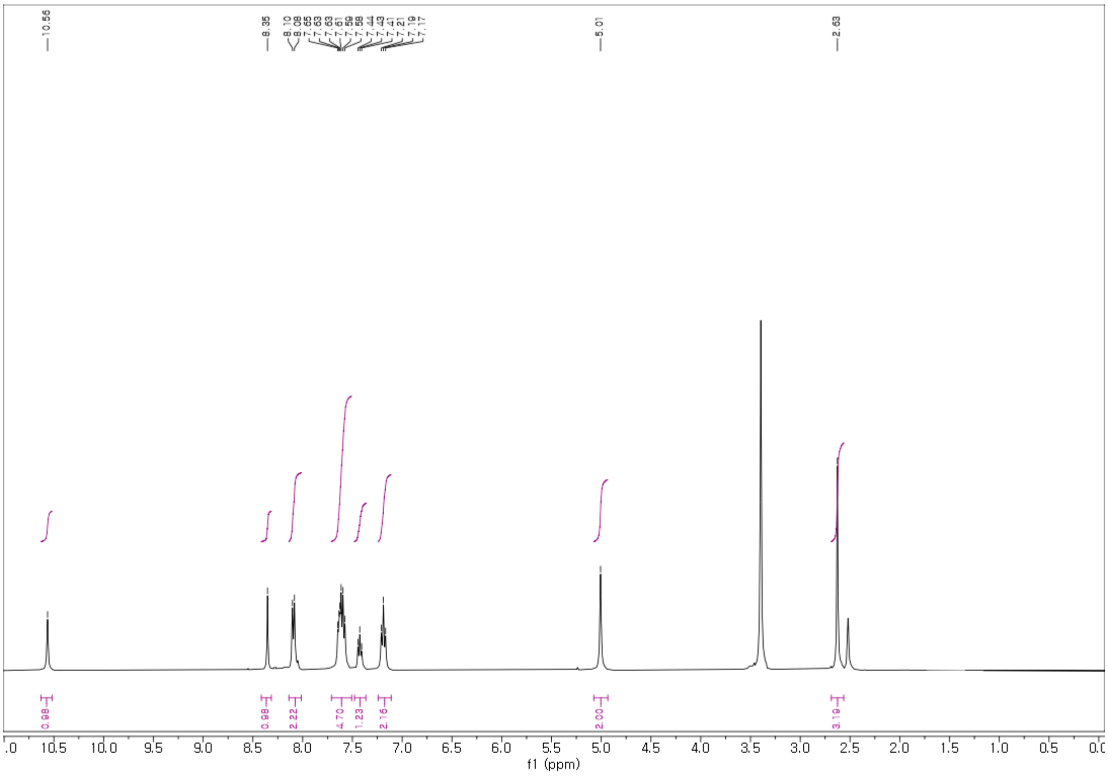

^{1}H-NMR spectra of compound 4a in DMSO-d_6

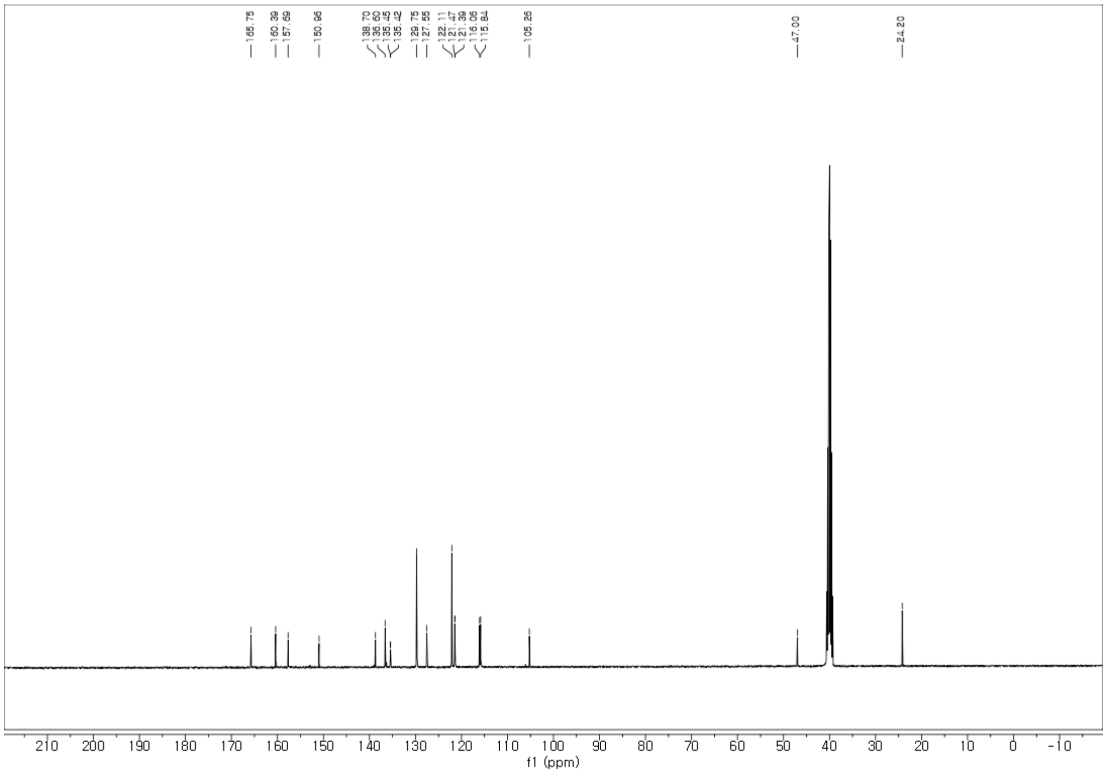

^{13}C-NMR spectra of compound 4a in DMSO-d_6

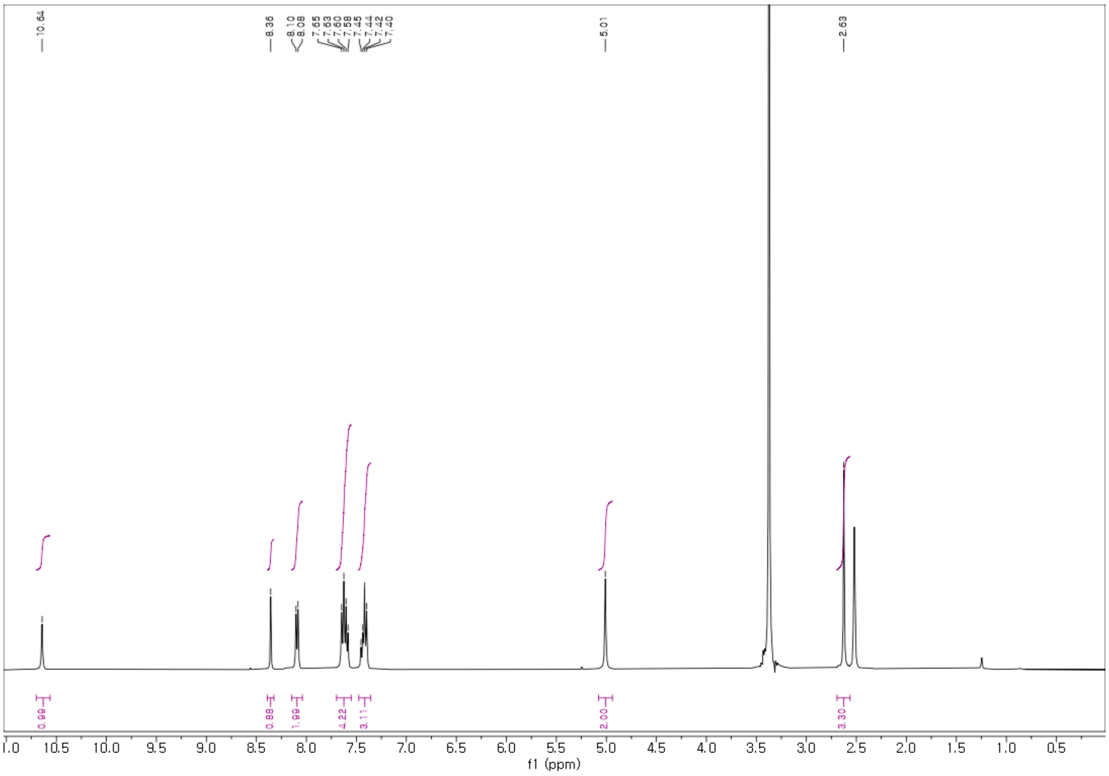

^{1}H-NMR spectra of compound 4b in DMSO-d_6

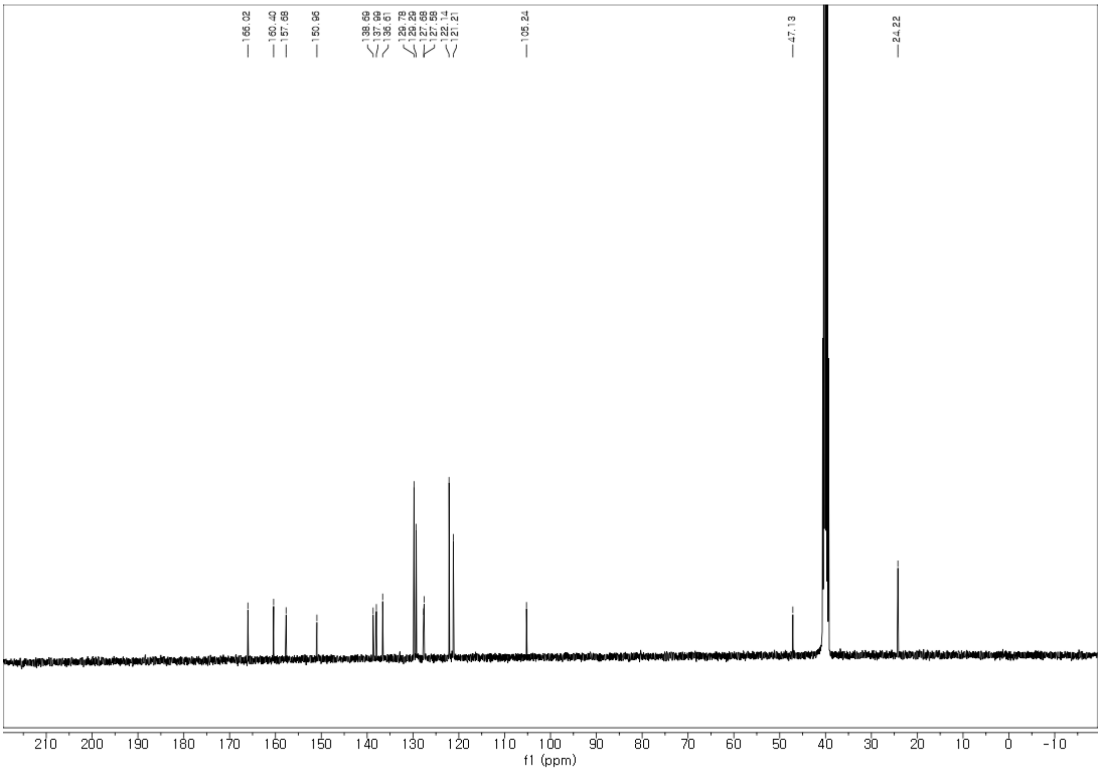

^{13}C-NMR spectra of compound 4b in DMSO-d_6

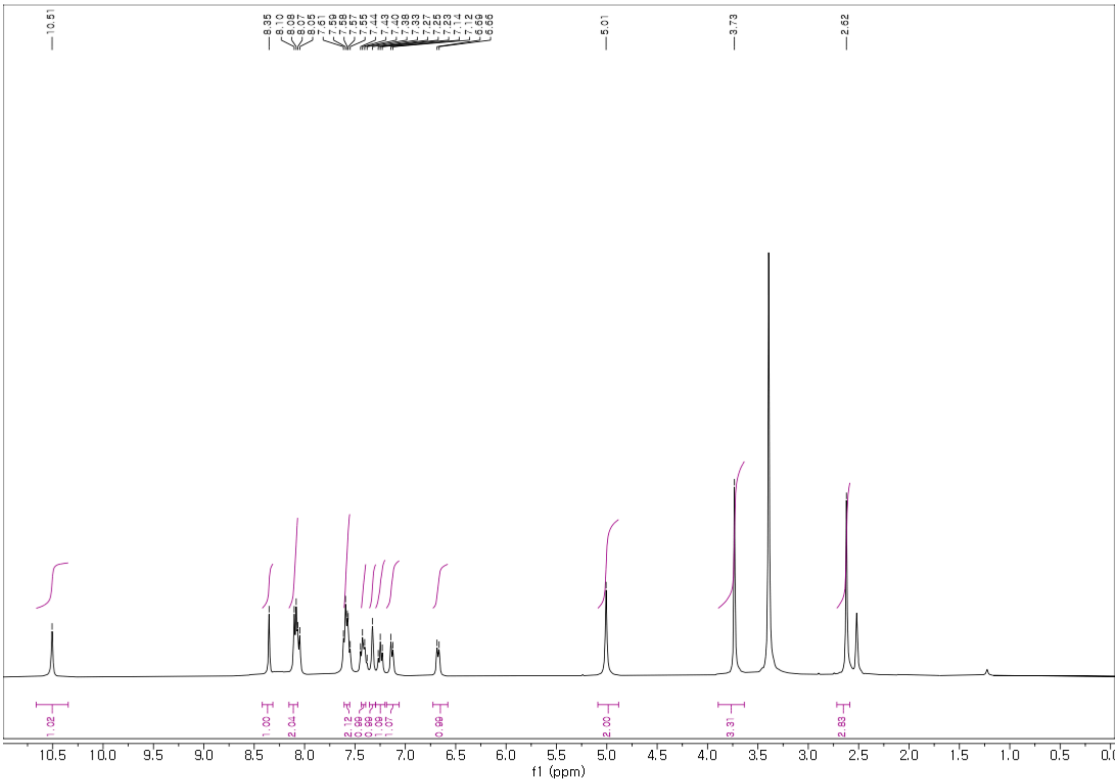

^{1}H-NMR spectra of compound 4c in DMSO-d_6

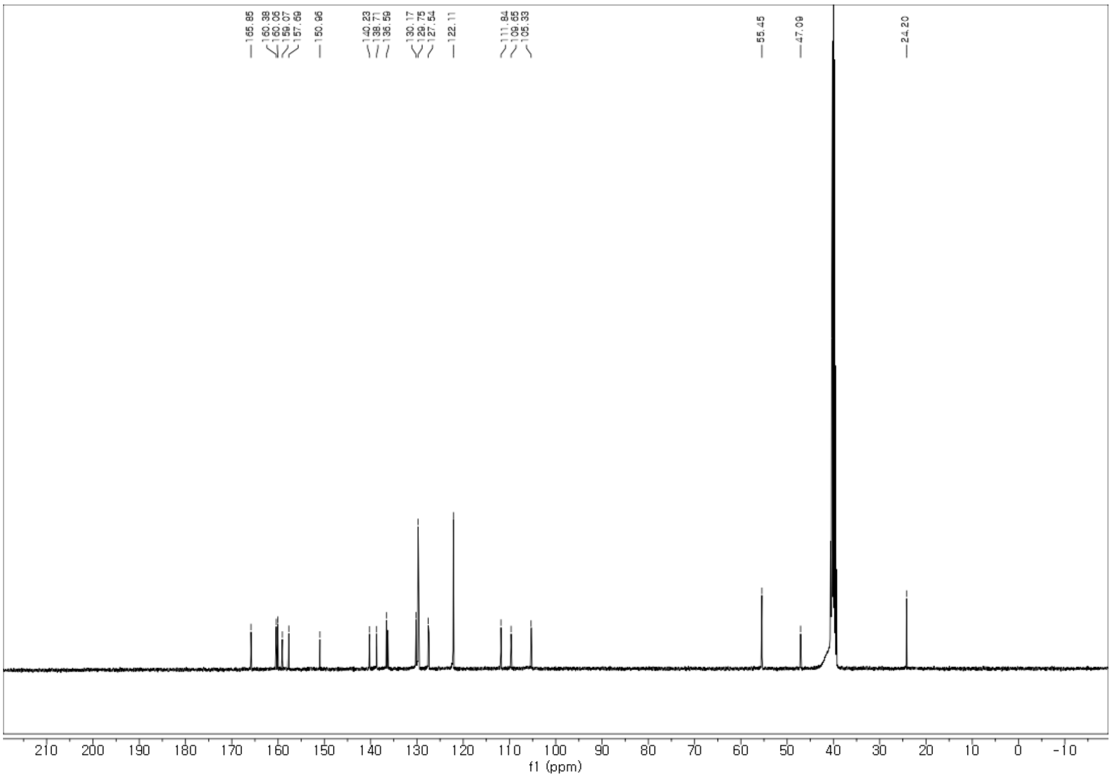

^{13}C-NMR spectra of compound 4c in DMSO-d_6

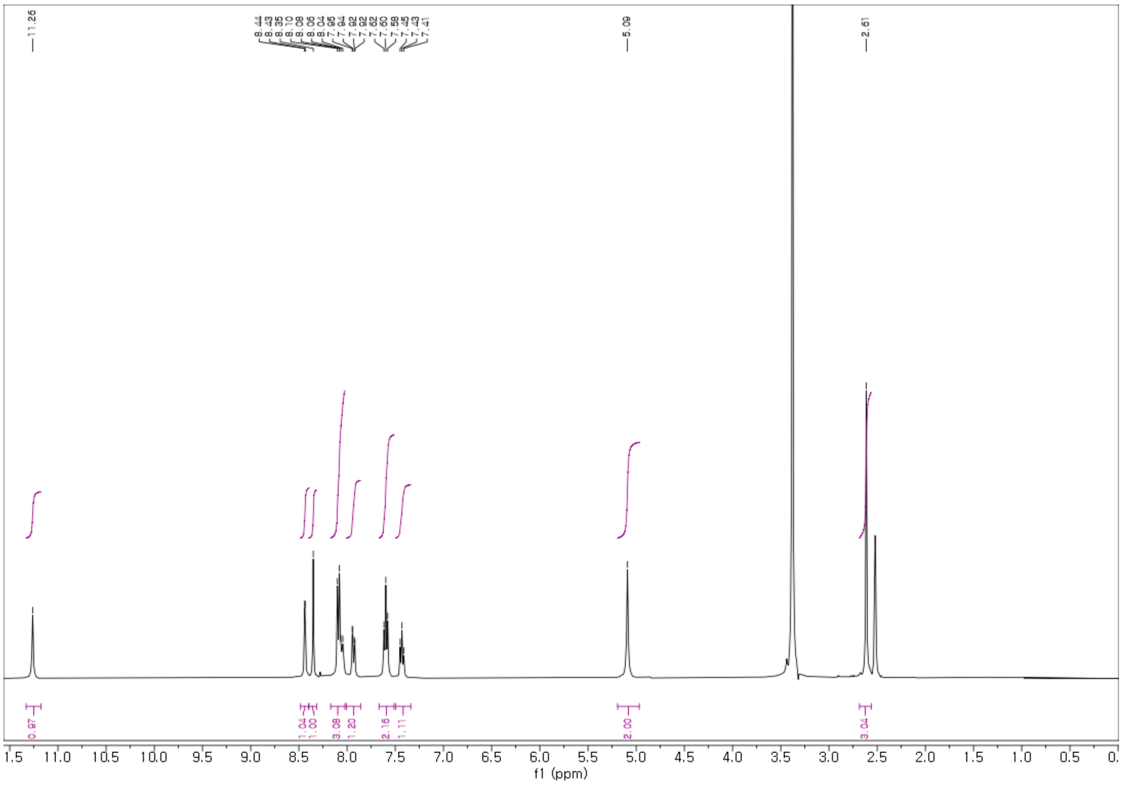

^{1}H-NMR spectra of compound 4d in DMSO-*d₆*

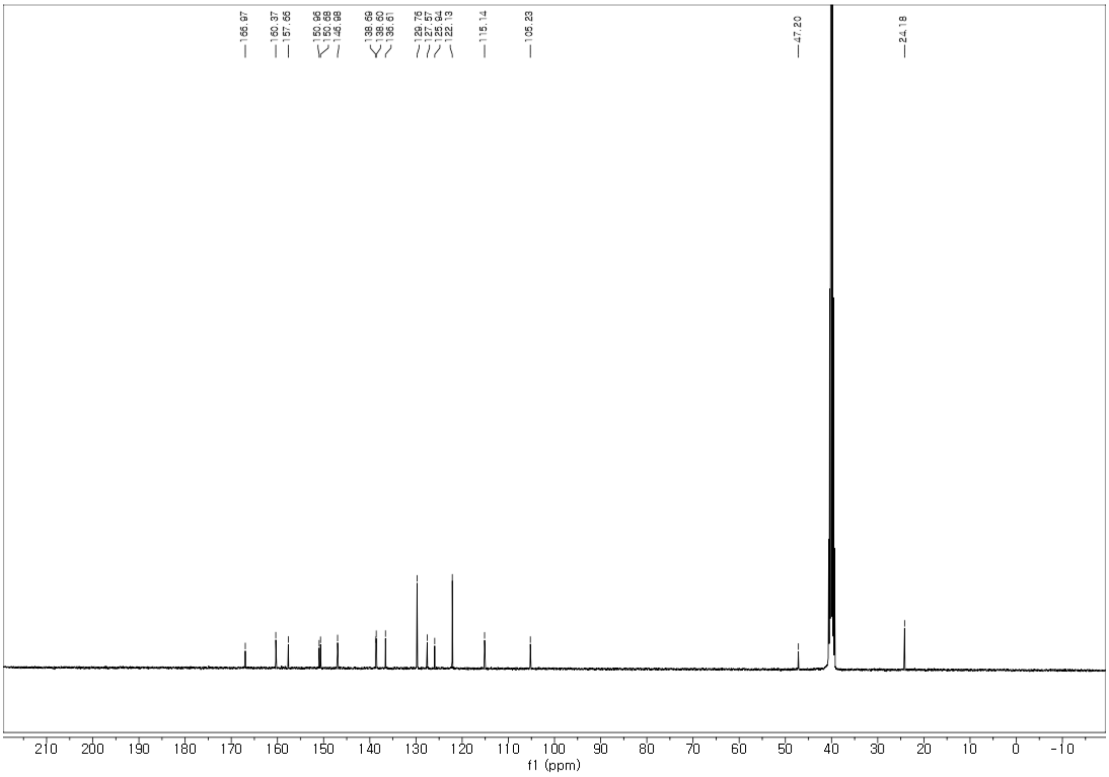

^{13}C-NMR spectra of compound 4d in DMSO-d_6

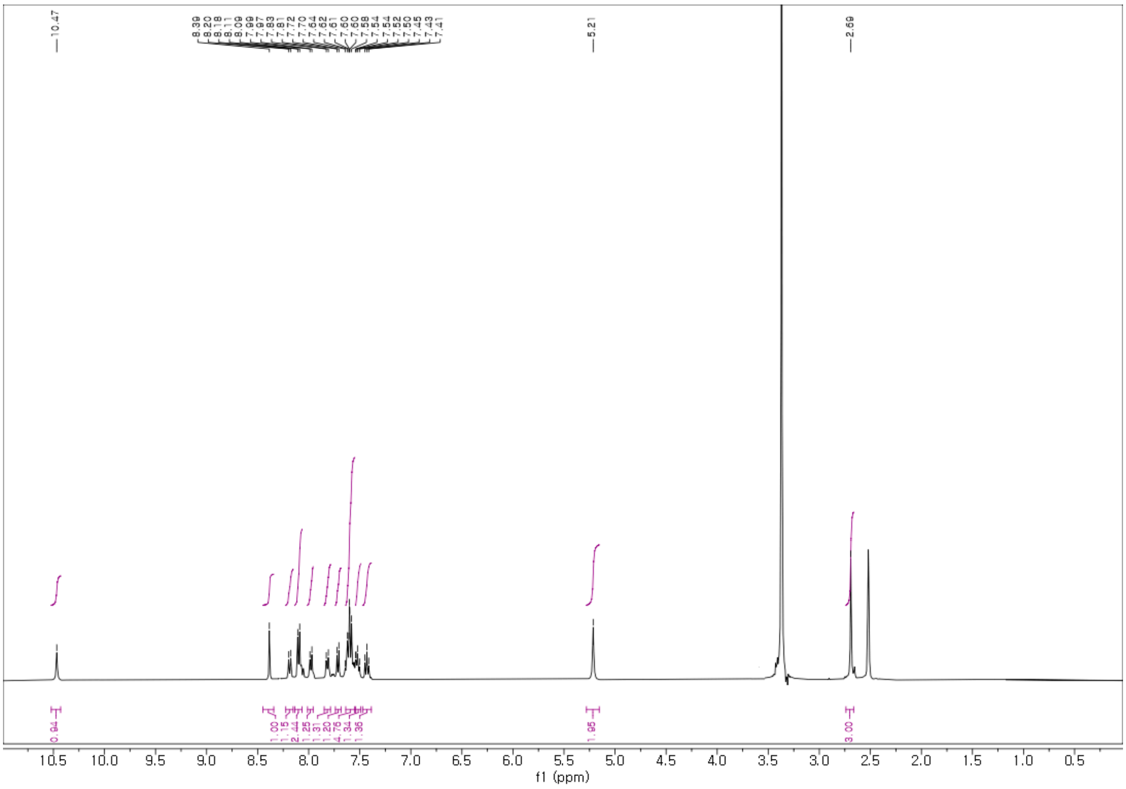

^{1}H-NMR spectra of compound 4e in DMSO-d_6

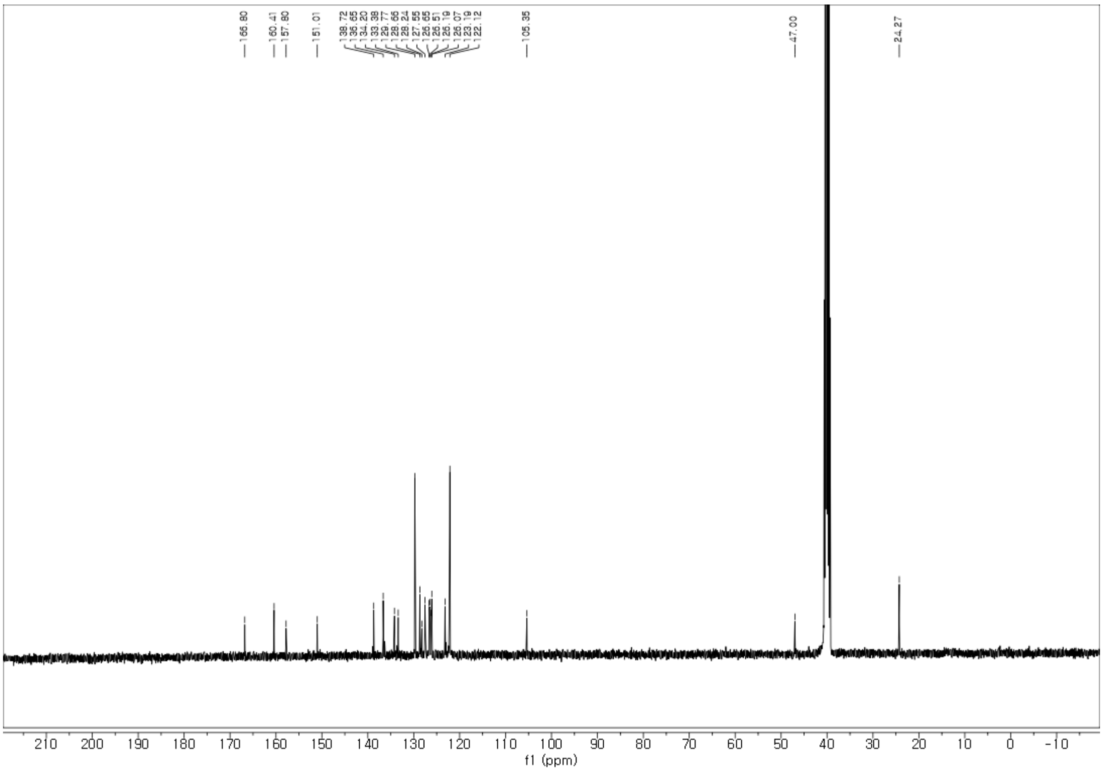

^{13}C-NMR spectra of compound 4e in DMSO-d_6
